# Supplementary material for: Ultra-high-field imaging reveals increased whole brain connectivity underpins cognitive strategies that attenuate pain
Source: eLife. 2020 Sep 2;9:e55028. doi: 10.7554/eLife.55028 (PMC7498261; doi:10.7554/eLife.55028)
Supplement: Transparent reporting form [file elife-55028-transrepform.docx]

*eLife’s* transparent reporting form

We encourage authors to provide detailed information *within their submission* to facilitate the interpretation and replication of experiments. Authors can upload supporting documentation to indicate the use of appropriate reporting guidelines for health-related research (see [EQUATOR Network](http://www.equator-network.org/%20)), life science research (see the [BioSharing Information Resource](https://biosharing.org/)), or the [ARRIVE guidelines](http://www.plosbiology.org/article/info:doi/10.1371/journal.pbio.1000412) for reporting work involving animal research. Where applicable, authors should refer to any relevant reporting standards documents in this form.

If you have any questions, please consult our Journal Policies and/or contact us: [editorial@elifesciences.org](mailto:editorial@elifesciences.org).

Sample-size estimation

- You should state whether an appropriate sample size was computed when the study was being designed
- You should state the statistical method of sample size computation and any required assumptions
- If no explicit power analysis was used, you should describe how you decided what sample (replicate) size (number) to use

Please outline where this information can be found within the submission (e.g., sections or figure legends), or explain why this information doesn’t apply to your submission:

A power analysis is computationally very demanding and the number of relevant brain regions is large.

Due to the fact that the critical effect size is unknown for the considered experimental conditions, for each selected region we have repeatedly generated random data according to our linear mixed model with the pre-specified values of the model parameters, where the true value of the effect of interest (the slope) was fixed to be equal to the value estimated from our original data. For each generated dataset we tested whether the observed effect (the slope) exceed the pre-specified threshold. The ratio of the number of threshold exceedances and the total number of repeated randomisations (10000) defined the power (the true effect size equal to the value estimated from the original data). The selected threshold had to be corrected for multiple comparisons as well. We used the very same threshold from the manuscript which is based on 5000 randomisations (Schulz et al., 2019) and the subsequent distribution of the most significant effect of an entire volume. This rather conservative statistical approach is implemented in PALM software package (Winkler et. al. 2014). The results suggest that the power is dependent on the respective brain region and experimental condition.

We have estimated the statistical power of the voxel-wise BOLD data with the suggested approach for each of the experimental conditions at several selected regions.

| x | y | z | Power: condition Counting | Power: condition imagination | Power condition reappraisal |
| --- | --- | --- | --- | --- | --- |
| 56 | 150 | 67 | 0.09 | 0.49 | 0.46 |
| 55 | 131 | 83 | 0.72 | 0.03 | 0.01 |
| 127 | 80 | 116 | 0.99 | 0 | 0 |
| 57 | 149 | 67 | 0.19 | 0.49 | 0.55 |
| 49 | 147 | 73 | 0.01 | 0.92 | 0.35 |
| 85 | 101 | 106 | 0.99 | 0.45 | 0.02 |

Replicates

- You should report how often each experiment was performed
- You should include a definition of biological versus technical replication
- The data obtained should be provided and sufficient information should be provided to indicate the number of independent biological and/or technical replicates
- If you encountered any outliers, you should describe how these were handled
- Criteria for exclusion/inclusion of data should be clearly stated
- High-throughput sequence data should be uploaded before submission, with a private link for reviewers provided (these are available from both GEO and ArrayExpress)

Please outline where this information can be found within the submission (e.g., sections or figure legends), or explain why this information doesn’t apply to your submission:

The experiment has been performed 22 times in 22 subjects. As stated in the manuscript, 2 subjects had to be excluded due to poor quality of the imaging data. The low quality was caused by excessive head movement of more than 2mm per direction. This is a standard procedure in neuroimaging and has been specified in the methods section.

None of the participants has been recorded twice.

Statistical reporting

- Statistical analysis methods should be described and justified
- Raw data should be presented in figures whenever informative to do so (typically when N per group is less than 10)
- For each experiment, you should identify the statistical tests used, exact values of N, definitions of center, methods of multiple test correction, and dispersion and precision measures (e.g., mean, median, SD, SEM, confidence intervals; and, for the major substantive results, a measure of effect size (e.g., Pearson's r, Cohen's d)
- Report exact p-values wherever possible alongside the summary statistics and 95% confidence intervals. These should be reported for all key questions and not only when the p-value is less than 0.05.

Please outline where this information can be found within the submission (e.g., sections or figure legends), or explain why this information doesn’t apply to your submission:

The required information is given in the “Results” and “Methods” section of the manuscript. The exact statistical coefficients (t-values) are specified in the supplementary spreadsheets.

(For large datasets, or papers with a very large number of statistical tests, you may upload a single table file with tests, Ns, etc., with reference to sections in the manuscript.)

Group allocation

- Indicate how samples were allocated into experimental groups (in the case of clinical studies, please specify allocation to treatment method); if randomization was used, please also state if restricted randomization was applied
- Indicate if masking was used during group allocation, data collection and/or data analysis

Please outline where this information can be found within the submission (e.g., sections or figure legends), or explain why this information doesn’t apply to your submission:

N/A

Additional data files (“source data”)

- We encourage you to upload relevant additional data files, such as numerical data that are represented as a graph in a figure, or as a summary table
- Where provided, these should be in the most useful format, and they can be uploaded as “Source data” files linked to a main figure or table
- Include model definition files including the full list of parameters used
- Include code used for data analysis (e.g., R, MatLab)
- Avoid stating that data files are “available upon request”

Please indicate the figures or tables for which source data files have been provided:

The statistical results that were used to generate the figures were submitted alongside the manuscript as spreadsheets.

The formula that has been used for the statistical analysis in Matlab has been specified in the manuscript. The code that indicates how the formula has to be integrated into the analysis is specified on the GitHub page of the co-author Viktor Witkovsky alongside further example code:

https://github.com/witkovsky/HPmixed.
